# Supplementary material for: Impact of COVID-19 Vaccination on Heart Rate Variability: A Systematic Review
Source: Vaccines (Basel). 2022 Dec 7;10(12):2095. doi: 10.3390/vaccines10122095 (PMC9787739; doi:10.3390/vaccines10122095)

Figure S1. Meta-analysis on RMSSD (ms) after vaccination data from Presby et al. (2022)

1-day after the dose

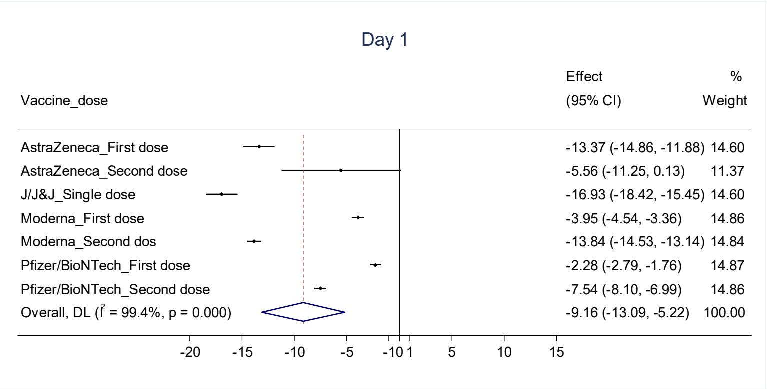

2-day after the dose

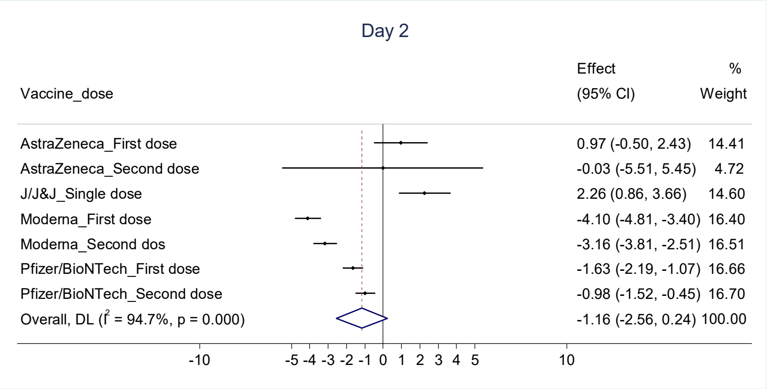

3-day after the dose

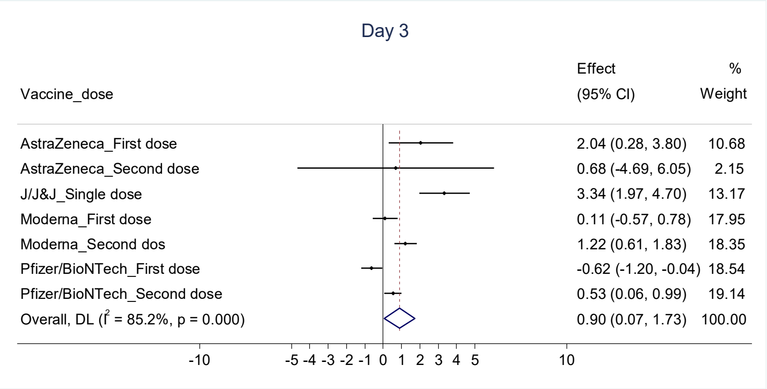

4-day after the dose

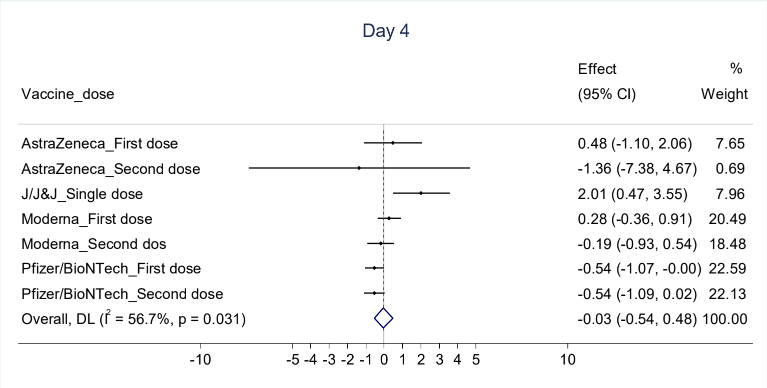

5-day after the dose

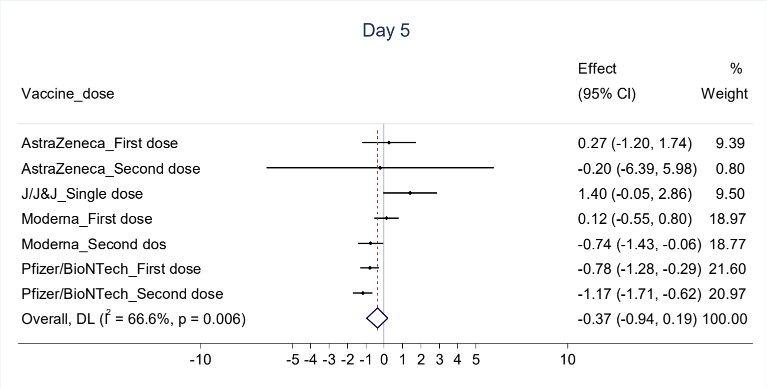

6-day after the dose

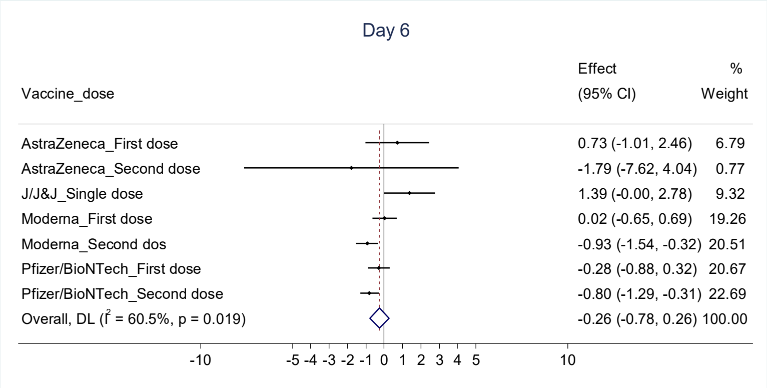

7-day after the dose

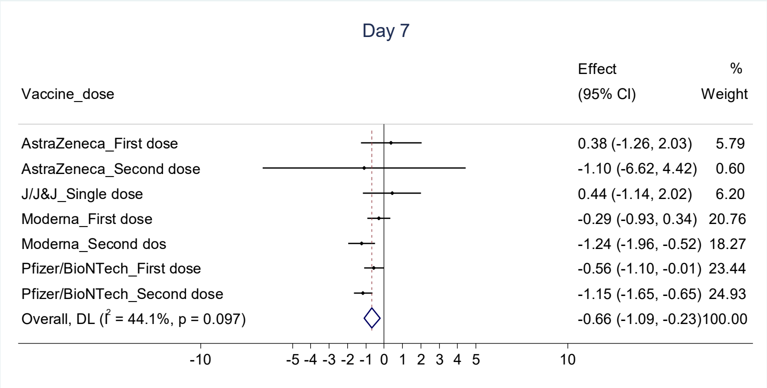

Supplement: Supplementary file 1 [file vaccines-10-02095-s001.zip › Figure S1.pdf]
